# PAI-1 Regulates the Invasive Phenotype in Human Cutaneous Squamous Cell Carcinoma

**DOI:** 10.1155/2009/963209

**Published:** 2010-03-01

**Authors:** Jennifer Freytag, Cynthia E. Wilkins-Port, Craig E. Higgins, J. Andrew Carlson, Agnes Noel, Jean-Michel Foidart, Stephen P. Higgins, Rohan Samarakoon, Paul J. Higgins

**Affiliations:** ^1^Center for Cell Biology & Cancer Research, Albany Medical College, 47 New Scotland Avenue, Albany, NY 12208, USA; ^2^Department of Pathology, Albany Medical College, 47 New Scotland Avenue, Albany, NY 12208, USA; ^3^Laboratory of Tumor and Developmental Biology, Groupe Interdisciplinaire de Génoprotéomique Appliqué-Cancer, University of Liège, Avenue de l'Hôpital 3, 4000 Liège, Belgium

## Abstract

The emergence of highly aggressive subtypes of human cutaneous squamous cell carcinoma (SCC) often reflects increased autocrine/paracrine TGF-*β* synthesis and epidermal growth factor receptor (EGFR) amplification. Cooperative TGF-*β*/EGFR signaling promotes cell migration and induces expression of both proteases and protease inhibitors that regulate stromal remodeling resulting in the acquisition of an invasive phenotype. In one physiologically relevant model of human cutaneous SCC progression, TGF-*β*1+EGF stimulation increases the production of several matrix metalloproteinases (MMPs), among the most prominent of which is MMP-10—an MMP known to be elevated in SCC in situ. Activation of stromal plasminogen appears to be critical in triggering downstream MMP activity. Paradoxically, PAI-1, the major physiological inhibitor of plasmin generation, is also upregulated under these conditions and is an early event in progression of incipient epidermal SCC. One testable hypothesis proposes that TGF-*β*1+EGF-dependent MMP-10 elevation directs focalized matrix remodeling events that promote epithelial cell plasticity and tissue invasion. Increased PAI-1 expression serves to temporally and spatially modulate plasmin-initiated pericellular proteolysis, further facilitating epithelial invasive potential. Defining the complex signaling and transcriptional mechanisms that maintain this delicate balance is critical to developing targeted therapeutics for the treatment of human cutaneous malignancies.

## 1. Epithelial Skin Cancer Initiation

 Nonmelanoma skin cancers (NMSCs) (i.e., basal cell, squamous cell, and Merkel cell carcinomas) are the most common human malignancies [1, 2]. In North America alone, >50% of all neoplasms arise in the skin [3] and the incidence of NMSC in Australia for the year 2002 was more than five times the incidence of all other cancers combined [4]. Relative to other cutaneous tumors, advanced squamous cell carcinoma (SCC) is aggressive, resistant to localized therapy with significant associated mortality and increasing in frequency [5].

 The emergence of epithelial skin tumors appears causally linked to ultraviolet (UV) radiation exposure. Specific UV-B “signature” base changes (C→T or CC→TT), particularly in codons 177 (basal cell carcinoma) and 278 (SCC) in the tumor suppressor p53 gene [6–8], likely occur early in epidermal carcinogenesis. Indeed, UV-associated p53 mutations are prevalent in solar radiation-induced actinic keratosis; 10% of these lesions progress to SCC and 60% of all SCC arise within actinic keratoses [9–11]. Transition of a normal keratinocyte to an initiated pre- or early malignant phenotype, in fact, often involves loss- or gain-of-function mutations in p53, with characteristic karyotypic changes including gains in chromosomes 7, 9, 18 (early on) and 3q, 8q, 9q, and 11q in later stages of tumor progression, *ras *gene mutation/activation/amplified expression (10–30% incidence), and inactivation of cell cycle inhibitors [7, 12–15]. While epidermal cancers associated with mutant *ras* expression may be cell type-dependent [16], molecular events that accompany the development of lesional subsets in both premalignant cutaneous lesions (actinic keratosis) and SCC [10, 11, 17] are similar. p53 gain-of-function versus loss-of-function mutations, moreover, may actually influence different stages in cutaneous SCC progression with gain-of-function changes associated with acceleration to SCC in the context of an oncogenic *ras* gene [14, 18]. At least one p53-activating gain-of-function mutation (p53^R172H^) results in increased skin tumor formation/progression and metastatic spread [18].

## 2. Epithelial Cell Plasticity and Tumor Progression

The accumulated genetic/epigenetic changes accompanying evolution of aggressive subtypes of cutaneous SCC are intertwined in a complex signaling landscape emanating from both tumor cells and stromal-derived elements (e.g., hepatocyte growth factor (HGF); epidermal growth factor (EGF); platelet-derived growth factor (PDGF); transforming growth factor-*β* (TGF-*β*)) [19–24]. TGF-*β*1 is a particularly robust initiator of epithelial “plasticity” (usually referred to as epithelial-to-mesenchymal transition or EMT), a likely facilitator of tumor invasion and metastasis (see, e.g., [22, 24]). The EMT “phenotome” however depends on physiologic context (i.e., embryogenesis, fibrosis/wound healing, tumor progression), the involved cell type, and the actual initiating stimulus [24].

 Elevated expression of transforming growth factor-*β*1 (TGF-*β*1) in the tumor microenvironment appears causally linked to creation of highly aggressive metastatic variants [19–23]. Acquired resistance to TGF-*β*1-mediated growth suppression, moreover, is frequently accompanied by mutation, allelic loss, or misregulation of elements within the TGF-*β*1 signaling network (e.g., TGF-*β*RI, TGF-*β*RII, SMAD2, SMAD4, the coreceptors endoglin, and betaglycan) (see, e.g., [25]). Such signaling defects, particularly in later stage tumors, are often coupled to constitutive epidermal growth factor receptor (EGFR) activation as a result of receptor amplification and/or autocrine ligand release [26–30]. The associated reprogramming of gene expression initiates and perpetuates TGF-*β*1-induced phenotypic plasticity [21, 31–37]. 

**Figure 1:**
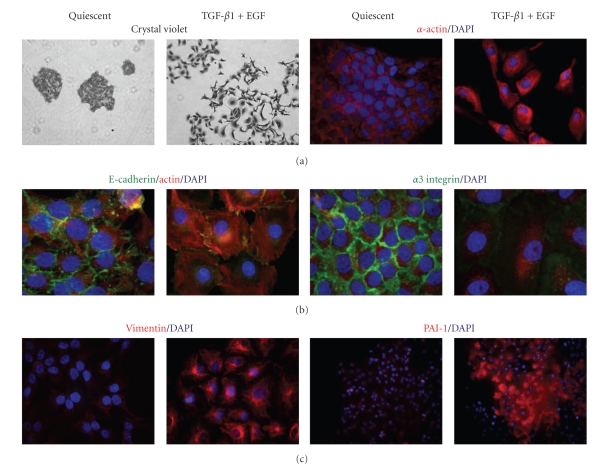
*Combination stimulation with TGF-*β*1+EGF induces a plastic response in HaCaT II-4 cells*. A model system was devised in which small colonies of HaCaT II-4 cells, seeded on tissue culture plastic, were serum-starved followed by addition of TGF-*β*1 (1 ng/mL) + EGF (10 ng/mL). The induced acquisition of a spindle-shaped, highly migratory phenotype, resulted in marked colony dispersal within 24–48 hours. Cell scattering was accompanied by the loss of E-cadherin (green) and *α*3 integrin (red) immunostaining at cell-cell junctions, and the gain of several mesenchymal markers, such as *α*-smooth muscle actin and vimentin with construction of a well-developed vimentin filament network. Induced PAI-1 expression (within 6 hours) was a prominent and early feature of growth factor-stimulated EMT.

**Figure 2:**
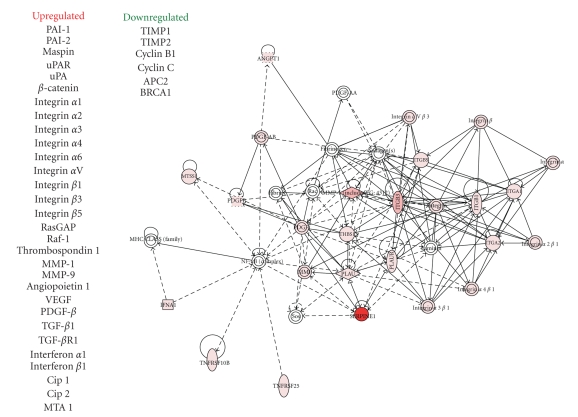
*Microarray transcript profiling and pathway analysis of TGF-*β*1+EGF-impacted genes in HaCaT II-4 keratinocytes*. Focused microarrays of dual growth factor-stimulated HaCaT II-4 cells revealed the increased expression of mRNAs encoding proteins involved in angiogenesis, stromal invasion, and control of pericellular proteolysis. PAI-1 transcripts were the most highly upregulated (>168-fold), induced early (within 6 hours) of stimulation and prior to the onset of colony dispersal. Ingenuity Pathway clustergram mapping describes potential functional interactions among the complement of induced genes. Pathway analyses of many of these genes (see also Table 1) indicate that several including uPA, uPAR, SERPINE1, and the MMPs are TGF-*β*1 targets and encode key elements in the integrative proteolytic cascades that regulate focalized stromal degradation and tumor invasion.

 Recent data mining of the actual repertoire of plasticity genes (i.e., the EMT transcriptome) has significantly enhanced our understanding of the biology of human cutaneous tumor progression while also providing a comparative map of expressed/repressed genes in actinic keratosis and SCC versus normal skin [38, 39]. Although the spectrum of likely candidate genes identified in different studies varies, plasminogen activator inhibitor type-1 (PAI-1; SERPINE1), the major physiologic regulator of the pericellular plasmin-generating cascade, has consistently emerged as a prominent member of the subset of TGF-*β*1-induced, EMT-associated genes in transformed human keratinocytes [34, 40]. PAI-1 is significantly increased in epithelial cells undergoing a mesenchymal-like conversion following activation of the E-cadherin transcriptional repressors, EMT-inducers, Snail, Slug, or E47 indicating that expression of this serine protease inhibitor is a general characteristic of the plastic phenotype [41]. Use of a novel, physiologically-relevant (i.e., p53 mutant, Ha-*ras*-expressing), dual growth factor (TGF-*β*1+EGF)-stimulated model of EMT in transformed human keratinocytes (HaCaT II-4 cells) (Figure 1) and microarray profiling defined PAI-1 as the most highly upregulated transcript of the early gene set (Figure 2; Table 1). The acquisition of a spindle-like, actively motile, behavior in this system was preceded by a decrease in E-cadherin immunoreactivity, the induction of vimentin and *α*-smooth muscle actin (Figure 1), and a genetic signature typical of an aggressive epithelial cell type (Table 1). Ingenuity Pathway analyses of many of these genes (Figure 2; Table 1) indicate that several (e.g., MMPs, uPA, uPAR, SERPINE1) are direct targets of TGF-*β*1, as well as NF-*κ*B, highlighting complex associations among EMT, the tumor microenvironment, and the attendant inflammatory response. Importantly, such clustergrams illustrate the highly coordinate and interdependent nature of the defined pericellular proteolytic cascades involved in focalized stromal degradation and tumor invasion (see, e.g., [34, 35, 38–41]). 

**Figure 3:**
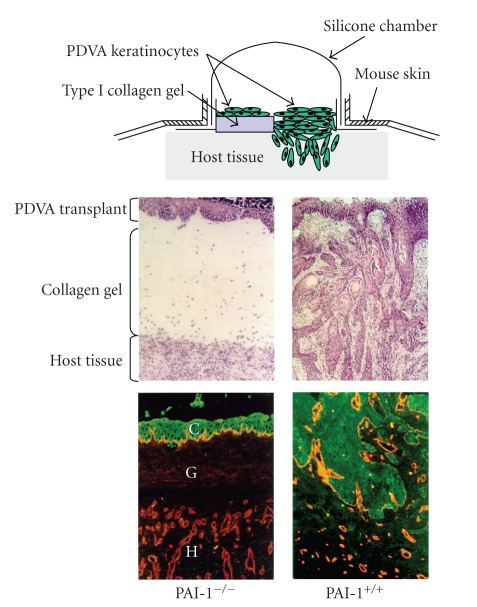
*Cutaneous carcinoma invasion and tumor angiogenesis are suppressed in PAI-1^−/−^ mice*. Malignant murine (PDVA) keratinocytes, cultured on a collagen gel in a silicone implantation chamber (top schematic), were transplanted onto PAI-1^−/−^ and wild-type PAI-1^+/+^ mice. Tumor implantation in PAI-1^−/−^ hosts resulted in a dramatic impairment of stromal invasion and failure to develop a supporting angiogenic network unlike the robust responses evident in wild-type animals. Tissue sections were stained with hematoxylin/eosin (two upper panels) or immunostained for keratin (green; to identify transplanted carcinoma cells) and type IV collagen (red; to delineate capillary vessel basement membrane (two lower panels)).

**Figure 4:**
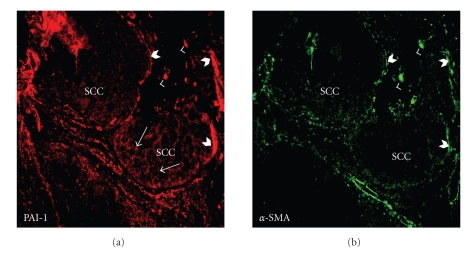
*In situ distribution of PAI-1 in an early invasive human cutaneous squamous cell carcinoma*. Sections were dually stained for PAI-1 (red; (a)) and *α*-smooth muscle actin (*α*-SMA, green; (b)). PAI-1 was evident in the SCC epithelium at the invasive front (arrows). Prominent PAI-1-expression also colocalized to *α*-SMA-positive cells, a marker for myofibroblasts, at the carcinoma periphery. Barbed arrowheads indicate PAI-1/*α*-SMA at the tumor perimeter while arrowheads depict PAI-1/*α*-SMA in stromal cells.

 Elevated PAI-1 tumor levels signal a poor prognosis and reduced disease-free survival in patients with various malignancies including breast, lung, ovarian, and oral SCC [42–46]. Current data suggest a model in which this SERPIN maintains an angiogenic “scaffold,” stabilizes nascent capillary vessel structure, and facilitates tumor cell stromal invasion through precise control of the peritumor proteolytic microenvironment [42, 47, 48]. Indeed, recent targeting of PAI-1 expression in endothelial cells and exogenous introduction of stable PAI-1 variants confirmed that PAI-1 is critical to nascent vessel stabilization and preservation of collagen matrix integrity [35, 49, 50]. In vivo studies, moreover, clearly implicate PAI-1 as an important, perhaps stage-dependent, determinant in cutaneous tumor invasion and the associated angiogenic response [47, 48, 51, 52] (Figure 3). PAI-1 likely “titrates” the extent and locale of collagen matrix remodeling, facilitating tumor invasion into the stroma while maintaining an angiogenic network by inhibiting capillary regression. Molecular knockdown “rescue” strategies, in fact, confirmed PAI-1 to be a positive regulator of keratinocyte migration and an inhibitor of plasminogen-induced anoikis [53]. PAI-1 upregulation is an early event in the progression of incipient epidermal SCC, often localizing to tumor cells and cancer-associated myofibroblasts at the invasive front [54–56] and, more importantly, is a marker with significant prognostic value [43–46]. Identification of PAI-1 (Figure 4(a)) in SCC-proximal *α*-SMA-positive stromal myofibroblasts (Figure 4(b)), furthermore, implies a more global involvement as a matricellular modulator of invasive potential [55–57] consistent with the increasing appreciation of the role of tumor stromal fibroblasts in cancer progression [58].

### 2.1. Stromal Remodeling Accompanies the Acquisition of Epithelial Plasticity

 Costimulation of human cutaneous SCC (HaCaT II-4) cells with TGF-*β*1+EGF promotes a plastic transition typical of late-stage tumor progression [35, 61] (Figure 1). This conversion to a more aggressive phenotype appears to be due, in part, to deregulated growth factor signaling and the transcriptional reprogramming that supports stromal remodeling events [62–69]. Plasmin generation, in particular, accompanies cooperative TGF-*β*1/EGFR signaling during the evolution of keratinocyte cell plasticity and is a critical event in the downstream activation of a complex and highly interdependent uPA-plasmin-matrix metalloproteinase (MMP) cascade [35, 70–80]. uPA, uPAR, and MMP expression levels are, in fact, significantly upregulated in HaCaT II-4 cells following stimulation with TGF-*β*1+EGF (e.g., Figure 2). The combination of TGF-*β*1+EGF, therefore, augments both matrix deposition, through TGF-*β*1-dependent upregulation of fibronectin, laminin, proteoglycans, tenascin, thrombospondin and PAI-1 production, and focal degradation by dependent increases in MMPs-1, -2, -3, -9, -10, -11, -13, and -21 [35, 66–71, 81–83].

TGF-*β*1- and/or EGF-stimulated synthesis of the generally epithelial-restricted MMP-10 (stromelysin-2) [72, 73], which targets a broad spectrum of matrix components including collagens types III, IV, and V, gelatin, elastin, fibronectin, proteoglycans and laminin, as well as proMMPs-1, -7, -8, -9, and -13 [74] is particularly significant. SCC of the head and neck, esophagus, oral cavity, and skin expresses elevated levels of MMP-10 [75–78]. While not detectable in intact skin, during cutaneous wound healing MMP-10 is expressed by keratinocytes that comprise the migrating tongue [79], where its activity appears to be important in stromal remodeling during cutaneous wound healing [79]. Despite an inability to cleave collagen type-I, a major dermal component, MMP-10, promotes plasmin-dependent collagenolysis by TGF-*β*1+EGF-stimulated HaCaT II-4 cells in a 3-dimensional system [35]. MMP-10, in fact, “superactivates” collagenase 1 (MMP-1), increasing MMP-1-dependent activity >10-fold compared to its activation by plasmin alone [72] creating a significant proteolytic axis within the cutaneous environment. 

**Figure 5:**
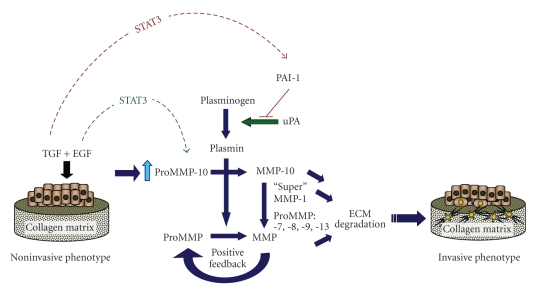
*Proposed mechanistic context for TGF-*β*1+EGF-enhanced plasmin-dependent collagen matrix remodeling and its contribution to development of an invasive phenotype*. Dual growth factor-stimulated HaCaT II-4 keratinocytes cultured on a three-dimensional collagen gel upregulate critical stromal remodeling factors that both disrupt and preserve matrix integrity. In the presence of active plasmin, increased MMP-10 promotes MMP activation and creates a proteolytic axis that accelerates collagen degradation through “superactivation” of MMP-1. STAT3 phosphorylation may serve as a temporal switch in this process, through its ability to both promote EGF-stimulated proMMP-10 expression and antagonize induction of TGF-*β*1 target genes (i.e., PAI-1, fibronectin) [59]. The synergistic upregulation of PAI-1 in response to TGF-*β*1+EGF may subsequently shift this proteolytic balance, enabling PAI-1 to “titrate” the extent and locale of collagen matrix remodeling to facilitate tumor cell stromal invasion. Indeed, PAI-1 induction is an early event in this phenotypic transition and required for the motile response since PAI-1 knockdown (with siRNA constructs) effectively inhibited TGF-*β*1+EGF-initiated colony scattering [60].

 Several MMPs, including MMP-10, are synergistically increased following costimulation of intestinal epithelial cells with TGF-*β*1+EGF [80]. In HaCaT II-4 keratinocytes, dual stimulation with TGF-*β*1+EGF induces MMP-10 expression while dramatically enhancing PAI-1 production and stromal invasion [35]. Since type-1 collagen degradation is essential for dermal remodeling, cutaneous tumor invasion may well be considerably dependent on MMP-10 activity. Indeed, MMP-10 upregulation, concomitant with increased STAT3 phosphorylation, accompanies the development of invasive behavior in breast cancer [81]. Similarly, EGF-dependent MMP-10 expression in bladder tumor cells is associated with changes in STAT3 signaling [82]. While the link between STAT3 activation and MMP-10 expression in cutaneous tumor progression remains to be determined, STAT3 over-expression/activation parallels invasive traits in cutaneous SCC [83, 84] suggesting that STAT3 may temporally regulate expression of proteolytically active components in the stromal microenvironment. Our studies indicate, moreover, that PAI-1 regulates MMP-10-dependent collagenolysis in TGF-*β*1+EGF-stimulated HaCaT II-4 keratinocytes [35]. Collectively, the current data suggest a model (Figure 5) in which MMP-10 induction in response to coincubation with TGF-*β*1+EGF activates MMPs-1, -7, -8, -9, and -13 stimulating plasmin-dependent matrix proteolysis. A corresponding upregulation of PAI-1 provides a sensitive focalized mechanism for titering the extent and duration of extracellular matrix degradation consequently sustaining a stromal scaffold necessary for tissue invasion. STAT3 in this context may promote this phenotype by regulating growth factor-dependent expression of critical remodeling factors such as MMP-10 and PAI-1 (Figure 5).

### 2.2. TGF-*β*1/EGFR Pathway Integration in PAI-1 Expression Control

**Figure 6:**
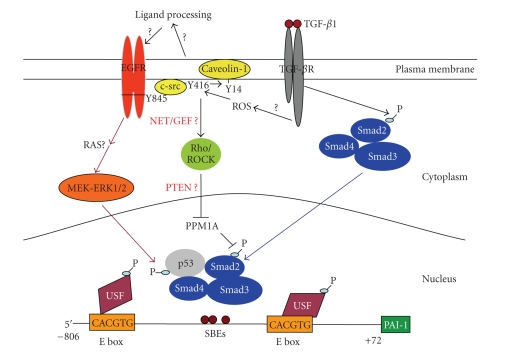
*A model for TGF-*β*1 induced PAI-1 transcription*. Emerging studies suggest that transcriptional outputs from both SMAD2/3 as well as non-SMAD (e.g., EGFR-MEK/ERK) pathways are absolutely critical for TGF-*β*1-mediated PAI-1 induction. Activated *Src* kinases (e.g., c-*Src*), downstream of TGF-*β*1 receptor, function as an upstream regulator of EGFR transactivation (by Y845 phosphorylation). c-*Src* also modulates Caveolin-1^Y14^ phosphorylation, and likely stimulates Rho/ROCK-dependent maintenance of SMAD2/3 transcriptional activity (by suppressing nuclear levels or activity of the SMAD2/3 phosphatase PPM1A). ERK1/2 (downstream of EGFR activation), or p38 kinases, may phosphorylate p53 and the bHLH-LZ upstream stimulatory factor proteins 1/2 (USF1/2) in response to TGF-*β*1. Indeed, SMAD2/3 appears to cooperate with p53 and USF family transcription factors for maximal TGF-*β*1-directed PAI-1 gene expression.

In several common carcinoma types, including cutaneous SCC, the combination of TGF-*β*1+EGF effectively initiates and maintains the dramatic morphological restructuring and genomic responses characteristic of the plastic phenotype [35, 61, 80]. In particular tumor models, the addition of EGF serves to activate the *ras *→*raf *→MEK→ERK cascade as a collateral stimulus to TGF-*β*R-dependent signaling. Clearly, cooperative, albeit complex, interactions between TGF-*β*1- and EGFR-activated pathways involving EGFR/pp60^c-src^, p21^ras^ and mitogen-activated extracellular kinase (MEK) [85] and the MAP kinases ERK/p38 appear mechanistically linked to epithelial tumor cell plasticity, at least in HaCaT II-4 cells [28, 30, 86–88]. The nonreceptor tyrosine kinase pp60^c-*src*^ is, in fact, a critical intermediate in a TGF-*β*1-initiated transduction cascade leading to MEK involvement, PAI-1 transcription, and downstream phenotypic responses [28, 85, 86, 88]. TGF-*β*1 complements EGF-mediated signaling to the MAPK/AKT pathways to effect EMT consistent with the requirement for oncogenic *ras* in TGF-*β*1-induced EMT [89, 90]. Disruption of TGF-*β*1-stimulated ERK1/2 phosphorylation and PAI-1 transcription by *src* family kinase inhibitors, as well as blockade of EGFR signaling with AG1478, suggests that pp60^c-*src*^, perhaps through phosphorylation of the Y845 *src*-kinase EGFR target residue, regulates MEK-ERK-dependent PAI-1 expression [28, 85, 91] (Figure 6). While the actual mechanism underlying TGF-*β*1-associated pp60^c-*src*^ kinase/EGFR stimulation remains to be determined, TGF-*β*1-dependent release of EGFR ligands (e.g., HB-EGF, amphiregulin and/or TGF-*α*) via MMP- or ADAM-dependent processes is one likely possibility for at least some cell types [60, 92]. Alternatively, the TGF-*β*1-stimulated formation of integrin/FAK/p130^cas^/EGFR complexes may initiate ligand-independent EGFR activation and pp60^c-*src*^ recruitment [91, 93, 94]. Indeed, in HaCaT cells, TGF-*β*1 transactivates the EGFR in a complex manner requiring *src* kinase signaling and production of reactive oxygen species but may not involve the shedding of EGFR ligands [30, 95]. The effective blockade of TGF-*β*1-stimulated ERK1/2 phosphorylation and PAI-1 transcription by *src* kinase-targeting pharmacologic agents, as well as the EGFR inhibitor AG1478, and the requirement for MEK-ERK signaling for the full inductive effect of TGF-*β*1, suggests that pp60^c-*src*^, perhaps through phosphorylation of the Y845 *src*-specific EGFR substrate residue regulates the MEK-ERK-dependent PAI-1 expression transduction cascade [28, 30, 51, 85, 86]. While specific mechanisms remain to be clarified, it is apparent that cooperative EGFR signaling is an essential aspect of TGF-*β*1-stimulated PAI-1 expression which provides novel insights as to the impact of TGF-*β*1 in late-stage human cutaneous tumor progression.

### 2.3. PAI-1 Transcription: Links to p53

Members of the p53 family are critical elements in a subset of TGF-*β*1 transcriptional responses due, at least in part, to the ability of MAP kinase-phosphorylated p53 to bind SMAD2 [96–100]. DNase I footprinting/methylation interference and oligonucleotide mobility shift analyses confirmed, moreover, that p53 binds to a recognition motif in the PAI-1 promoter resulting in both p53 sequence-driven reporter gene transcription and induced expression of the endogenous PAI-1 gene [101]. Two p53 half-sites (AcACATGCCT, cAGCAAGTCC) [Profile Hidden Markov Model score= 82; 89] likely regulate p53-dependent PAI-1 gene activation [102]. p53-mediated PAI-1 expression control, moreover, is likely to involve nontranscriptional mechanisms as well since p53 binds to a 70 nt sequence on the PAI-1 mRNA 3′ UTR resulting in increased mRNA stabilization [103].

 p53 is also required for maximal PAI-1 expression in response to TGF-*β*1 since induced transcription is significantly attenuated in p53 siRNA knockdown cells [98] as well as in p53^−/−^ mouse fibroblasts (Samarakoon and Higgins, unpublished data). This is consistent with the observation that p53-deficient lung tumor cells synthesize little or no PAI-1 while vector-engineered introduction of wild-type p53 rescues both basal and inducible PAI-1 expressions [103]. The recent analysis of the upstream region of the PAI-1 gene provides some insight as to the possible mechanisms underlying p53-dependent PAI-1 gene control. The PAI-1 promoter PE2 region hexanucleotide E box (CACGTG), a site juxtaposed to 3 SMAD-binding elements, is occupied by upstream stimulatory factor (USF) in response to TGF-*β*1 stimulation [85, 104]. Phasing analysis revealed that certain bHLH-LZ members of the MYC family (including USF) orient DNA bending toward the minor groove [105] which could potentially promote interactions between p53, bound to its downstream half-site motif, with SMAD2 tethered to the upstream PE2 region SMAD-binding elements, thus, providing a molecular basis for SMAD2/p53 complex formation and subsequent transcriptional activation of the PAI-1 gene (Figure 6).

### 2.4. Implications on Cell Growth Control

p53 mutations occur in 40–60% of all skin cancers [9, 106] suggesting that direct p53 transcriptional targets (such as the PAI-1 gene) may be activated, repressed, or dysregulated as a consequence of p53 mutation with associated loss or gain of function. Indeed, p53 is a major element in PAI-1 induction in response to TGF-*β*1 [98] and may be critical particularly in the setting of increased autocrine TGF-*β*1 expression during cutaneous SCC progression. The role of PAI-1 in subsequent tumor progression, however, may be more complex than previously appreciated. Ectopic expression of wild-type PAI-1 in breast cancer cells or in p53-deficient murine and human fibroblasts, in fact, initiates a senescence-like growth arrest [92, 107] while RNAi-mediated PAI-1 knockdown (PAI-1^KD^) or PAI-1 genetic deficiency (PAI-1^−/−^ genotype) results in escape from replicative senescence in primary mouse and human fibroblasts [107]. Proliferation of PAI-1^−/−^ endothelial cells, and PAI-1^KD^ fibroblasts appears due, at least in part, to sustained activation of the PI3K-AKT-GSK3*β* pathway, increased AKT^Ser473^ phosphorylation, nuclear retention of cyclin D1 [107, 108] and, perhaps, increased inactivation of the tumor suppressor PTEN [108]. Importantly, PAI-1^−/−^ mouse embryo fibroblasts (MEFs), PAI-1^KD^ HaCaT keratinocytes, and PAI-1^KD^ MEFs are resistant to TGF-*β*1-initiated growth inhibition although PAI-1 deficiency does not interfere with canonical TGF-*β*1 signaling such as SMAD phosphorylation or *p21^CIP1^* and *p15^INK4B^* induction [109].

 Collectively, these data suggest a multifunctional relationship between PAI-1 expression and tumor progression. Elevated PAI-1 levels may inhibit (at least transiently) tumor cell proliferation while stimulating migration and stromal invasion by providing a sensitive focalized mechanism for titering the extent and duration of extracellular matrix degradation, sustaining a stromal scaffold necessary for tissue invasion. This carefully orchestrated process may also serve to promote tumor cell survival by preventing anoikis during the precarious process of cell detachment and readhesion to a new, likely foreign, tissue microenvironment. Importantly, these findings underscore the potential diversity of new molecular targets that can be exploited for therapeutic benefit. Refining the current understanding of PAI-1 gene regulation, and relevant signaling pathways, may lead to the discovery of critical regulatory factors that ultimately prove important in stage-specific treatment of human cutaneous malignancies.

## Figures and Tables

**Table 1:** Transcript levels for select Cancer Pathway genes.

Gene name	Symbol	Quiescent versus TGF-*β*1+EGF
Angiopoietin 1	ANGPT1	3.01
Breast cancer 1, early onset	BRCA1	−3.18
Cyclin-dependent kinase 2	CDK2	2.46
Cyclin-dependent kinase inhibitor 1A (p21, Cip1)	CDKN1A	7.41
Interferon *α*1	IFN*α*1	5.66
Interferon *β*1, fibroblast	IFN*β*1	6.87
Integrin *α*1	ITG*α*1	5.66
Integrin *α*2	ITG*α*2	18.25
Integrin *β*1	ITG*β*1	11.71
Integrin *β*3	ITG*β*3	59.30
Integrin *β*5	ITG*β*5	5.82
Matrix metallopeptidase 1	MMP1	59.30
Matrix metallopeptidase 9	MMP9	55.33
Metastasis associated 1	MTA1	2.19
Metastasis associated 1 family, member 2	MTA2	1.82
Metastasis suppressor 1	MTSS1	5.58
Platelet-derived growth factor *β* polypeptide	PDGFB	9.51
Plasminogen activator, urokinase	PLAU	2.64
Plasminogen activator, urokinase receptor	PLAUR	8.00
Serpin peptidase inhibitor, clade E (plasminogen activator inhibitor-1)	SERPINE1	168.90
Transforming growth factor *β*1	TGF*β*1	5.54
Transforming growth factor *β* receptor 1	TGF-*β*R1	3.46
Thrombospondin 1	THBS1	9.25
Tumor necrosis factor receptor superfamily, member 10b	TNFRSF10B	2.16
Tumor necrosis factor receptor superfamily, member 25	TNFRSF25	3.53
Vascular endothelial growth factor A	VEGFA	23.26

## Abbreviations

SCC: Squamous cell carcinoma
EGF: Epidermal growth factor
EGFR: Epidermal growth factor receptor
TGF-*β*1: Transforming growth factor-*β*1
MMP: Matrix metalloproteinase
NMSC: Nonmelanoma skin cancer
UV: Ultraviolet
TGF-*β*R: TGF-*β* receptor
EMT: Epithelial-to-mesenchymal transition
PAI-1: Plasminogen activator inhibitor type-1
SERPINE1: Serine protease inhibitor, clade E, member 1
uPA: Urokinase plasminogen activator
uPAR: Urokinase plasminogen activator receptor
STAT3: Signal transducer and activators of transcription protein 3
SMAD: Sma/Mad homologues
ERK: Extracellular signal-regulated kinases
MEK: Mitogen-activated protein kinase/ERK kinase
FAK: Focal adhesion kinase
MEFs: Mouse embryo fibroblasts.
